# 4032 An exploration of the perceptions of young women with breast cancer with varying health literacy levels about the usefulness of cancer educational materials

**DOI:** 10.1017/cts.2020.200

**Published:** 2020-07-29

**Authors:** Pearman Parker, Jean C McSweeney, Kristin Zorn, Kristie Hadden, Carolyn Greene, L. Joseph Su, Cathy Meade

**Affiliations:** 1 University of Arkansas Translational Research Institute; 2 University of Arkansas for Medical Sciences; 3 Moffitt Cancer Center

## Abstract

OBJECTIVES/GOALS: Young women (18 – 45 years of age) with breast cancer have complex medical and psychosocial needs. Educational materials are often used as tools in patient-centered communication. However, these materials disseminate complex health information in print-heavy formats and can be difficult to understand for women with varying health literacy levels. METHODS/STUDY POPULATION: In the first phase of this study, the principal investigator (PI) will recruit 40 diverse women to participate in four focus groups (FG) to explore the perceived usefulness of the most frequently used cancer educational materials. The PI will also obtain demographics and heath literacy levels of the FG participants using the Newest Vital Sign. In the second phase, the PI will assess the literacy demands of the ten most frequently used cancer educational print materials and five most frequently used websites described by the FG participants. The perceptions of the usefulness of materials and the literacy demands will then be used to appraise the effectiveness of materials within patient-centered cancer communication. RESULTS/ANTICIPATED RESULTS: Results from this study will provide a patient-centered blueprint that will be used to design more effective educational materials that treatment centers can incorporate into their patient-centered cancer communication process. The next step of this research will be to determine providers’ perceptions of cancer education materials used to exchange information within the patient-centered communication process. This will complement the patient findings and inform the development of the provider aspect of a communication intervention centered on designing educational materials for women with various health literacy levels within the patient-centered cancer communication process. DISCUSSION/SIGNIFICANCE OF IMPACT: Detecting the usefulness of cancer educational materials, as perceived by young women with breast cancer, is foundational to developing communication interventions that improve cancer outcomes. This study will identify how materials can be improved in the critical informational-exchange component of the patient-communication process.

